# Supplementary material for: A Likelihood Approach for Real-Time Calibration of Stochastic Compartmental Epidemic Models
Source: PLoS Comput Biol. 2017 Jan 17;13(1):e1005257. doi: 10.1371/journal.pcbi.1005257 (PMC5240920; doi:10.1371/journal.pcbi.1005257)
Supplement: S1 File — (TAR.GZ) [file pcbi.1005257.s014.tar.gz › HSPH_Online-SI-Revision/PF08/n10-extreme/n10-extreme_PF2_table8.pdf]

1	newly 3 weeks spec	33.	102.72	{87., 120.}	211.273	0.486854	newly 3 weeks cumul	135.	414.32	{377., 449.}	206.904	0.483048	newly 5 weeks spec	7.	56.29	{44., 70.}	704.143	0.894602	newly 5 weeks cumul	159.	544.77	{493., 594.}	242.623	0.530373	newly 50 weeks cumul	163.	713.87	{656., 782.}	337.957	0.635698
2	newly 3 weeks spec	108.	159.95	{137., 179.}	48.1019	0.167945	newly 3 weeks cumul	418.	589.56	{529., 646.}	41.0431	0.14768	newly 5 weeks spec	44.	105.83	{88., 120.}	140.523	0.37697	newly 5 weeks cumul	535.	827.6	{755., 907.}	54.6916	0.187752	newly 46 weeks cumul	683.	1265.68	{1131., 1402.}	85.3119	0.265613
3	newly 3 weeks spec	12.	65.67	{55., 76.}	447.25	0.734582	newly 3 weeks cumul	49.	280.64	{264., 295.}	472.735	0.757492	newly 5 weeks spec	3.	34.2	{27., 41.}	1040.	1.0521	newly 5 weeks cumul	60.	363.27	{347., 379.}	505.45	0.781757	newly 52 weeks cumul	67.	450.8	{427., 468.}	572.836	0.827557
4	newly 3 weeks spec	57.	107.46	{88., 132.}	88.5263	0.269851	newly 3 weeks cumul	214.	409.82	{351., 483.}	91.5047	0.278653	newly 5 weeks spec	34.	68.3	{49., 89.}	100.882	0.293703	newly 5 weeks cumul	292.	563.22	{472., 680.}	92.8836	0.281322	newly 46 weeks cumul	396.	804.49	{657., 986.}	103.154	0.301952
5	newly 3 weeks spec	61.	91.44	{77., 107.}	49.9016	0.172296	newly 3 weeks cumul	281.	365.34	{333., 394.}	30.0142	0.113031	newly 5 weeks spec	29.	49.34	{38., 60.}	70.1379	0.223629	newly 5 weeks cumul	366.	483.14	{441., 518.}	32.0055	0.119556	newly 49 weeks cumul	445.	638.81	{578., 689.}	43.5528	0.15581
6	newly 3 weeks spec	52.	111.62	{94., 129.}	114.654	0.328568	newly 3 weeks cumul	218.	451.87	{396., 494.}	107.28	0.314916	newly 5 weeks spec	24.	64.75	{53., 79.}	169.792	0.42598	newly 5 weeks cumul	283.	600.15	{518., 658.}	112.067	0.324775	newly 50 weeks cumul	342.	794.46	{671., 868.}	132.298	0.364146
7	newly 3 weeks spec	45.	109.24	{78., 138.}	142.756	0.376094	newly 3 weeks cumul	222.	442.25	{338., 523.}	99.2117	0.293248	newly 5 weeks spec	26.	62.11	{40., 79.}	138.885	0.363724	newly 5 weeks cumul	276.	588.44	{437., 691.}	113.203	0.32201	newly 51 weeks cumul	350.	783.18	{547., 947.}	123.766	0.340748
8	newly 3 weeks spec	74.	124.45	{105., 140.}	68.1757	0.223571	newly 3 weeks cumul	355.	484.71	{433., 518.}	36.538	0.134245	newly 5 weeks spec	40.	76.91	{64., 91.}	92.275	0.280233	newly 5 weeks cumul	452.	659.78	{592., 705.}	45.969	0.163311	newly 48 weeks cumul	577.	921.17	{808., 993.}	59.6482	0.201818
9	newly 3 weeks spec	75.	116.27	{99., 134.}	55.0267	0.186748	newly 3 weeks cumul	323.	455.98	{393., 504.}	41.1703	0.148021	newly 5 weeks spec	31.	70.1	{56., 83.}	126.129	0.349584	newly 5 weeks cumul	408.	615.6	{522., 693.}	50.8824	0.176787	newly 48 weeks cumul	485.	851.88	{708., 958.}	75.6454	0.242402
10	newly 3 weeks spec	97.	135.16	{115., 153.}	39.3402	0.141672	newly 3 weeks cumul	429.	533.05	{489., 582.}	24.2541	0.0933056	newly 5 weeks spec	65.	84.1	{68., 102.}	30.0308	0.109561	newly 5 weeks cumul	590.	725.77	{665., 801.}	23.0119	0.0887324	newly 48 weeks cumul	747.	1012.09	{906., 1129.}	35.4873	0.130374
11	newly 3 weeks spec	96.	157.21	{139., 176.}	63.7604	0.212832	newly 3 weeks cumul	365.	575.5	{542., 605.}	57.6712	0.19734	newly 5 weeks spec	43.	104.93	{89., 119.}	144.023	0.384264	newly 5 weeks cumul	474.	813.19	{774., 850.}	71.5591	0.234098	newly 43 weeks cumul	637.	1273.36	{1184., 1342.}	99.8995	0.300316
12	newly 3 weeks spec	56.	142.21	{120., 163.}	153.946	0.401118	newly 3 weeks cumul	258.	550.71	{504., 609.}	113.453	0.327851	newly 5 weeks spec	30.	87.78	{72., 106.}	192.6	0.461357	newly 5 weeks cumul	326.	748.98	{686., 831.}	129.748	0.359741	newly 48 weeks cumul	370.	1047.99	{954., 1163.}	183.241	0.450138
13	newly 3 weeks spec	46.	114.4	{97., 133.}	148.696	0.39229	newly 3 weeks cumul	235.	427.78	{394., 488.}	82.034	0.258428	newly 5 weeks spec	40.	75.43	{61., 91.}	88.575	0.270301	newly 5 weeks cumul	321.	594.77	{542., 687.}	85.2866	0.266167	newly 44 weeks cumul	406.	892.35	{798., 1062.}	119.791	0.339632
14	newly 3 weeks spec	50.	95.58	{71., 118.}	91.16	0.273812	newly 3 weeks cumul	229.	378.1	{303., 447.}	65.1092	0.212731	newly 5 weeks spec	25.	56.1	{38., 72.}	124.56	0.338858	newly 5 weeks cumul	291.	506.32	{402., 594.}	73.9931	0.234877	newly 49 weeks cumul	352.	686.21	{532., 817.}	94.9744	0.283031
15	newly 3 weeks spec	41.	116.33	{96., 134.}	183.732	0.449492	newly 3 weeks cumul	175.	448.84	{416., 501.}	156.48	0.407787	newly 5 weeks spec	20.	75.59	{62., 91.}	277.95	0.571707	newly 5 weeks cumul	229.	616.	{566., 700.}	168.996	0.428283	newly 46 weeks cumul	253.	893.04	{796., 1030.}	252.98	0.545602
16	newly 3 weeks spec	68.	108.7	{91., 122.}	59.8529	0.200572	newly 3 weeks cumul	284.	439.2	{400., 477.}	54.6479	0.187893	newly 5 weeks spec	35.	60.2	{48., 74.}	72.	0.230849	newly 5 weeks cumul	368.	579.59	{516., 634.}	57.4973	0.195718	newly 49 weeks cumul	425.	766.74	{682., 843.}	80.4094	0.25436
17	newly 3 weeks spec	58.	93.42	{72., 112.}	61.3448	0.201288	newly 3 weeks cumul	228.	381.11	{310., 427.}	67.1535	0.21961	newly 5 weeks spec	27.	51.96	{35., 66.}	92.8148	0.274281	newly 5 weeks cumul	296.	503.55	{410., 571.}	70.1182	0.226933	newly 51 weeks cumul	355.	651.46	{502., 726.}	83.5099	0.258348
18	newly 3 weeks spec	58.	94.48	{78., 105.}	62.8966	0.209507	newly 3 weeks cumul	249.	378.77	{349., 409.}	52.1165	0.181306	newly 5 weeks spec	33.	51.74	{44., 61.}	56.7879	0.191823	newly 5 weeks cumul	331.	501.31	{468., 534.}	51.4532	0.179582	newly 49 weeks cumul	406.	661.96	{617., 710.}	63.0443	0.211563
19	newly 3 weeks spec	19.	107.95	{94., 121.}	468.158	0.752471	newly 3 weeks cumul	129.	435.21	{400., 468.}	237.372	0.527219	newly 5 weeks spec	10.	59.47	{48., 72.}	494.7	0.769002	newly 5 weeks cumul	154.	573.75	{527., 617.}	272.565	0.570347	newly 51 weeks cumul	172.	746.96	{690., 801.}	334.279	0.636771
20	newly 3 weeks spec	82.	127.23	{112., 143.}	55.1585	0.188345	newly 3 weeks cumul	349.	502.76	{452., 546.}	44.0573	0.157332	newly 5 weeks spec	51.	76.97	{63., 93.}	50.9216	0.174165	newly 5 weeks cumul	464.	680.53	{614., 743.}	46.6659	0.165043	newly 48 weeks cumul	599.	935.64	{835., 1043.}	56.2003	0.192035
21	newly 3 weeks spec	71.	135.24	{117., 155.}	90.4789	0.27728	newly 3 weeks cumul	319.	508.2	{452., 548.}	59.3103	0.200961	newly 5 weeks spec	24.	88.36	{74., 103.}	268.167	0.56231	newly 5 weeks cumul	400.	706.74	{634., 765.}	76.685	0.245898	newly 45 weeks cumul	509.	1060.96	{936., 1163.}	108.44	0.317407
22	newly 3 weeks spec	15.	66.46	{57., 76.}	343.067	0.643699	newly 3 weeks cumul	86.	287.98	{271., 307.}	234.86	0.524198	newly 5 weeks spec	10.	32.49	{23., 40.}	224.9	0.502908	newly 5 weeks cumul	114.	367.2	{347., 391.}	222.105	0.507374	newly 52 weeks cumul	122.	444.03	{415., 474.}	263.959	0.560216
23	newly 3 weeks spec	49.	139.05	{113., 162.}	183.776	0.448037	newly 3 weeks cumul	175.	521.82	{441., 578.}	198.183	0.47194	newly 5 weeks spec	26.	92.34	{75., 112.}	255.154	0.544	newly 5 weeks cumul	236.	727.84	{612., 804.}	208.407	0.486438	newly 47 weeks cumul	286.	1091.4	{910., 1234.}	281.608	0.577906
24	newly 3 weeks spec	62.	109.56	{96., 123.}	76.7097	0.244869	newly 3 weeks cumul	297.	412.11	{379., 437.}	38.7576	0.141244	newly 5 weeks spec	38.	69.18	{55., 80.}	82.0526	0.255794	newly 5 weeks cumul	391.	567.45	{528., 603.}	45.1279	0.160737	newly 45 weeks cumul	543.	825.04	{761., 889.}	51.9411	0.180371
25	newly 3 weeks spec	17.	115.67	{101., 128.}	580.412	0.83087	newly 3 weeks cumul	104.	455.67	{433., 480.}	338.144	0.641266	newly 5 weeks spec	9.	71.13	{59., 84.}	690.333	0.89408	newly 5 weeks cumul	127.	616.45	{592., 641.}	385.394	0.685861	newly 48 weeks cumul	139.	849.59	{813., 884.}	511.216	0.785933
26	newly 3 weeks spec	8.	75.68	{62., 89.}	846.	0.971654	newly 3 weeks cumul	30.	311.25	{287., 339.}	937.5	1.0148	newly 5 weeks spec	1.	41.97	{32., 50.}	4097.	1.61674	newly 5 weeks cumul	36.	410.26	{383., 440.}	1039.61	1.05572	newly 51 weeks cumul	40.	528.75	{492., 570.}	1221.88	1.11978
27	newly 3 weeks spec	65.	114.45	{96., 134.}	76.0769	0.242274	newly 3 weeks cumul	303.	464.71	{408., 520.}	53.3696	0.184164	newly 5 weeks spec	45.	67.02	{53., 82.}	48.9778	0.167818	newly 5 weeks cumul	394.	620.93	{549., 695.}	57.5964	0.195827	newly 49 weeks cumul	475.	827.63	{720., 936.}	74.2379	0.238899
28	newly 3 weeks spec	25.	65.54	{56., 76.}	162.16	0.415376	newly 3 weeks cumul	120.	286.28	{264., 310.}	138.567	0.37625	newly 5 weeks spec	9.	33.31	{24., 42.}	270.111	0.558338	newly 5 weeks cumul	146.	366.35	{335., 393.}	150.925	0.397981	newly 52 weeks cumul	160.	448.06	{403., 483.}	180.038	0.445107
29	newly 3 weeks spec	36.	111.91	{96., 126.}	210.861	0.490199	newly 3 weeks cumul	170.	435.63	{407., 475.}	156.253	0.4078	newly 5 weeks spec	12.	69.49	{56., 82.}	479.083	0.757758	newly 5 weeks cumul	201.	590.56	{552., 642.}	193.811	0.467077	newly 47 weeks cumul	219.	825.03	{765., 904.}	276.726	0.574698
30	newly 3 weeks spec	50.	125.09	{105., 141.}	150.18	0.395465	newly 3 weeks cumul	199.	479.13	{436., 518.}	140.769	0.3804	newly 5 weeks spec	23.	77.67	{66., 90.}	237.696	0.524338	newly 5 weeks cumul	254.	655.52	{597., 707.}	158.079	0.410522	newly 47 weeks cumul	304.	927.44	{829., 1017.}	205.079	0.482675
31	newly 3 weeks spec	53.	83.97	{58., 107.}	60.0566	0.195951	newly 3 weeks cumul	252.	346.04	{258., 429.}	38.754	0.137511	newly 5 weeks spec	36.	46.99	{31., 62.}	37.1389	0.135168	newly 5 weeks cumul	334.	455.85	{337., 562.}	38.3683	0.136142	newly 50 weeks cumul	377.	595.91	{412., 776.}	58.7772	0.191928
32	newly 3 weeks spec	76.	146.89	{115., 172.}	93.2763	0.281629	newly 3 weeks cumul	281.	524.89	{424., 583.}	86.7936	0.268541	newly 5 weeks spec	36.	101.67	{77., 119.}	182.417	0.444526	newly 5 weeks cumul	374.	748.76	{606., 837.}	100.203	0.298425	newly 42 weeks cumul	533.	1225.73	{948., 1383.}	129.968	0.357456
33	newly 3 weeks spec	8.	73.78	{65., 83.}	822.25	0.962608	newly 3 weeks cumul	52.	303.02	{288., 321.}	482.731	0.765092	newly 5 weeks spec	2.	40.57	{33., 50.}	1928.5	1.30155	newly 5 weeks cumul	59.	401.	{388., 415.}	579.661	0.832065	newly 50 weeks cumul	62.	516.36	{499., 538.}	732.839	0.92036
34	newly 3 weeks spec	8.	56.32	{40., 80.}	604.	0.831622	newly 3 weeks cumul	61.	238.31	{179., 339.}	290.672	0.581609	newly 5 weeks spec	7.	28.46	{17., 43.}	306.571	0.582817	newly 5 weeks cumul	76.	306.78	{234., 453.}	303.658	0.594538	newly 52 weeks cumul	80.	382.16	{277., 586.}	377.7	0.664878
35	newly 3 weeks spec	95.	116.63	{97., 139.}	23.0632	0.0864869	newly 3 weeks cumul	347.	449.12	{399., 525.}	29.4294	0.109638	newly 5 weeks spec	52.	71.67	{54., 94.}	38.2885	0.132829	newly 5 weeks cumul	464.	611.84	{538., 743.}	31.8621	0.117263	newly 47 weeks cumul	569.	868.55	{740., 1096.}	52.645	0.179221
36	newly 3 weeks spec	110.	135.95	{117., 152.}	23.9909	0.0913814	newly 3 weeks cumul	389.	512.47	{472., 552.}	31.7404	0.118775	newly 5 weeks spec	47.	87.98	{74., 100.}	87.1915	0.268508	newly 5 weeks cumul	515.	711.81	{658., 765.}	38.2155	0.13962	newly 45 weeks cumul	717.	1053.08	{964., 1116.}	46.8731	0.165786
37	newly 3 weeks spec	60.	101.29	{83., 123.}	68.8167	0.222043	newly 3 weeks cumul	245.	407.29	{359., 488.}	66.2408	0.217412	newly 5 weeks spec	22.	59.74	{46., 79.}	171.545	0.425326	newly 5 weeks cumul	316.	545.	{483., 661.}	72.4684	0.23294	newly 48 weeks cumul	370.	739.36	{646., 910.}	99.827	0.295956
38	newly 3 weeks spec	10.	97.84	{85., 110.}	878.4	0.988627	newly 3 weeks cumul	32.	397.78	{382., 416.}	1143.06	1.09421	newly 5 weeks spec	1.	52.89	{44., 62.}	5189.	1.71964	newly 5 weeks cumul	36.	520.37	{501., 541.}	1345.47	1.15983	newly 51 weeks cumul	37.	671.01	{649., 692.}	1713.54	1.25841
39	newly 3 weeks spec	14.	77.44	{52., 98.}	453.143	0.73084	newly 3 weeks cumul	85.	318.65	{238., 386.}	274.882	0.565168	newly 5 weeks spec	6.	41.12	{26., 56.}	585.333	0.818219	newly 5 weeks cumul	101.	415.73	{309., 507.}	311.614	0.605515	newly 51 weeks cumul	112.	533.37	{384., 660.}	376.223	0.666802
40	newly 3 weeks spec	73.	121.09	{104., 138.}	65.8767	0.216875	newly 3 weeks cumul	282.	477.11	{436., 531.}	69.1879	0.227093	newly 5 weeks spec	30.	73.96	{60., 88.}	146.533	0.386621	newly 5 weeks cumul	357.	644.65	{580., 713.}	80.5742	0.255268	newly 48 weeks cumul	439.	894.37	{791., 998.}	103.729	0.307273
41	newly 3 weeks spec	66.	107.04	{92., 121.}	62.1818	0.206964	newly 3 weeks cumul	240.	416.14	{374., 455.}	73.3917	0.237705	newly 5 weeks spec	31.	64.61	{52., 77.}	108.419	0.313872	newly 5 weeks cumul	309.	564.53	{500., 620.}	82.6958	0.26035	newly 47 weeks cumul	381.	784.72	{679., 892.}	105.963	0.311514
42	newly 3 weeks spec	69.	115.91	{97., 132.}	67.9855	0.221645	newly 3 weeks cumul	344.	426.63	{394., 460.}	24.0203	0.0919002	newly 5 weeks spec	67.	79.13	{68., 92.}	19.6866	0.0755267	newly 5 weeks cumul	474.	598.53	{549., 649.}	26.2722	0.0996688	newly 42 weeks cumul	655.	936.93	{830., 1022.}	43.0427	0.153078
43	newly 3 weeks spec	15.	100.47	{79., 121.}	569.8	0.82029	newly 3 weeks cumul	71.	415.18	{359., 493.}	484.761	0.763276	newly 5 weeks spec	6.	56.08	{42., 73.}	834.667	0.96026	newly 5 weeks cumul	88.	545.72	{469., 648.}	520.136	0.788288	newly 52 weeks cumul	93.	701.54	{586., 851.}	654.344	0.872432
44	newly 3 weeks spec	89.	154.73	{134., 176.}	73.8539	0.237962	newly 3 weeks cumul	399.	560.73	{526., 593.}	40.5338	0.1471	newly 5 weeks spec	51.	105.16	{90., 119.}	106.196	0.311992	newly 5 weeks cumul	518.	793.34	{749., 834.}	53.1544	0.184479	newly 43 weeks cumul	705.	1252.74	{1157., 1335.}	77.6936	0.248785
45	newly 3 weeks spec	84.	118.22	{102., 134.}	40.7381	0.14595	newly 3 weeks cumul	323.	454.55	{416., 503.}	40.7276	0.147212	newly 5 weeks spec	39.	71.84	{57., 89.}	84.2051	0.259686	newly 5 weeks cumul	423.	617.83	{565., 687.}	46.0591	0.163234	newly 47 weeks cumul	539.	862.8	{776., 965.}	60.0742	0.202573
46	newly 3 weeks spec	113.	132.69	{110., 152.}	18.6814	0.0721911	newly 3 weeks cumul	424.	497.55	{439., 545.}	18.2335	0.0714997	newly 5 weeks spec	54.	85.39	{69., 102.}	58.2037	0.194373	newly 5 weeks cumul	553.	689.59	{607., 748.}	24.9421	0.094842	newly 45 weeks cumul	782.	1017.58	{865., 1123.}	30.1714	0.111956
47	newly 3 weeks spec	79.	135.77	{115., 166.}	71.8608	0.231548	newly 3 weeks cumul	324.	523.28	{478., 618.}	61.5062	0.206393	newly 5 weeks spec	43.	85.13	{68., 105.}	97.9767	0.291383	newly 5 weeks cumul	419.	716.4	{650., 836.}	70.9785	0.231162	newly 47 weeks cumul	531.	1020.62	{907., 1224.}	92.2072	0.281118
48	newly 3 weeks spec	58.	106.82	{92., 123.}	84.1724	0.262777	newly 3 weeks cumul	234.	430.31	{396., 470.}	83.8932	0.263612	newly 5 weeks spec	22.	60.68	{48., 71.}	175.818	0.435075	newly 5 weeks cumul	292.	570.1	{533., 623.}	95.2397	0.289614	newly 50 weeks cumul	344.	744.89	{694., 806.}	116.538	0.334609
49	newly 3 weeks spec	22.	96.86	{86., 107.}	340.273	0.64195	newly 3 weeks cumul	126.	398.53	{381., 417.}	216.294	0.499542	newly 5 weeks spec	10.	52.6	{44., 64.}	426.	0.716339	newly 5 weeks cumul	151.	522.33	{507., 543.}	245.914	0.538468	newly 51 weeks cumul	165.	674.05	{654., 702.}	308.515	0.610479
50	newly 3 weeks spec	52.	116.69	{103., 131.}	124.404	0.349384	newly 3 weeks cumul	223.	470.78	{448., 488.}	111.112	0.324282	newly 5 weeks spec	29.	68.33	{59., 80.}	135.621	0.368844	newly 5 weeks cumul	274.	629.31	{606., 649.}	129.675	0.360939	newly 49 weeks cumul	323.	841.17	{818., 873.}	160.424	0.415547
